# Integrating mechanical sensor readouts into organ-on-a-chip platforms

**DOI:** 10.3389/fbioe.2022.1060895

**Published:** 2022-12-16

**Authors:** Ingrid Anaya Morales, Christina-Marie Boghdady, Benjamin E. Campbell, Christopher Moraes

**Affiliations:** ^1^ Division of Experimental Medicine, McGill University, Montreal, QC, Canada; ^2^ Department of Chemical Engineering, McGill University, Montreal, QC, Canada; ^3^ Department of Biomedical Engineering, McGill University, Montreal, QC, Canada

**Keywords:** mechanobiology, organ-on-a-chip, biomechanics, tissue engineering, sensors

## Abstract

Organs-on-a-chip have emerged as next-generation tissue engineered models to accurately capture realistic human tissue behaviour, thereby addressing many of the challenges associated with using animal models in research. Mechanical features of the culture environment have emerged as being critically important in designing organs-on-a-chip, as they play important roles in both stimulating realistic tissue formation and function, as well as capturing integrative elements of homeostasis, tissue function, and tissue degeneration in response to external insult and injury. Despite the demonstrated impact of incorporating mechanical cues in these models, strategies to measure these mechanical tissue features in microfluidically-compatible formats directly on-chip are relatively limited. In this review, we first describe general microfluidically-compatible Organs-on-a-chip sensing strategies, and categorize these advances based on the specific advantages of incorporating them on-chip. We then consider foundational and recent advances in mechanical analysis techniques spanning cellular to tissue length scales; and discuss their integration into Organs-on-a-chips for more effective drug screening, disease modeling, and characterization of biological dynamics.

## 1 Introduction

Organs-on-a-chip (OoC) are microengineered cell and tissue culture platforms that drive cells to emulate highly realistic phenotypes, by recreating various features of the organ microenvironment (Leung et al., 2022). These microengineered tissues can then be used for drug testing, disease modeling, toxicology, and personalized medicine (Ingber, 2022). Studies over the last decade have suggested strong potential for these models in to capture relevant physiological or disease processes in a format that can be easily imaged and manipulated. Various OoCs have been developed to recreate the lung, blood vessel, heart, liver, gut, muscle, blood-brain barrier, skin barrier, amongst many others (Zhang et al., 2018); and have also been interconnected to evaluate organ-organ interactions in multi-organs on-a-chip and body-on-a-chip systems (Skardal et al., 2016; Ronaldson-Bouchard and Vunjak-Novakovic, 2018; Vogt, 2022).

The creation of a physiological or pathophysiological biomechanical environment is essential to successful recapitulation *in vivo* behavior in OoCs models (Clarke et al., 2021; Fuchs et al., 2021). *In vivo*, cells are exposed to biochemical and biomechanical stimuli critical for tissue development and cell response (Moraes et al., 2011), and recreating biomechanical cues such as substrate stiffness, geometric confinement, topography, stretch, compression, fluid shear stress, interstitial fluid flow, and hydrostatic pressure will likely continue to be essential components of OoC development (Zhang et al., 2018). As illustrative examples, lung-on-a-chip models incorporate cyclic mechanical strain mimicking the breathing motions which is essential in obtaining a physiological inflammatory response (Huh et al., 2012; Stucki et al., 2015; Huang et al., 2019; Zhu et al., 2022); Intestine-on-a-chip models incorporate peristalsis-like mechanical stretch to create a robust barrier to bacterial infection (Kasendra et al., 2018; Jalili-Firoozinezhad et al., 2019); and including shear stress in heart-on-a-chip and kidney-on-a-chip models alters inflammatory responses and drug uptake respectively (Lee et al., 2018; Ferrell et al., 2019).

Conversely, cells actively respond to these mechanical cues to further modulate the local mechanical microenvironment, in a tissue maturation feedback loop driven by the initial mechanical culture environment (Ergir et al., 2018). Monitoring these tissue features can provide important information about the state of development of the model, as well as the ways in which the model responds to toxicity and biochemical or biomechanical injury (Thompson et al., 2020). Recently, considerable focus has been placed on integrating biosensors directly into OoCs, to allow data collection in a non-invasive and continuous manner, with the potential to multiplex measurements over extended periods of time (Clarke et al., 2021; Fuchs et al., 2021). Biosensors have been developed to measure biochemical signals as metabolite concentration and barrier integrity, while other platforms quantify mechanics as stiffness and forces (Adam Kratz et al., 2019; Ferrari et al., 2020). Despite this focus, and the broad variety of electrochemical, optical, mechanical, acoustic, bead-based, and magnetic sensors that have been developed, demonstrations of their integration into OoCs have been relatively limited (Kilic et al., 2018; Rothbauer and Ertl, 2020). In this review, we briefly select sensors that have been integrated into OoCs and categorize these advances based on their utility for on-a-chip studies. We then consider foundational and recent advances in mechanical analysis techniques spanning cellular to tissue length scales and discuss their integration into OoCs for more effective drug screening, quantitative disease modeling, and characterization of biological dynamics in tissue fate and function.

## 2 Specific benefits of integrated sensors in the OoC context

Sensors and measurement tools play an essential role in interrogating all biological systems, and are often integrated into microfluidics to enhance their sensitivity, limits of detection, and utility in precious sample processing (Young and Moraes, 2015). However, some unique advantages arise from integrating sensing technologies directly into OoCs. In this section we identify and highlight the impact of these core measurement advantages using selected examples of OoC-sensor integrations.

### 2.1 Real-time measurements

In contrast with standard animal models, OoCs offer the promise of visualizing realistic cell behaviour and complex biological processes. The OoC structure allows both slow processes such as leukocyte infiltration (Gjorevski et al., 2020), or rapid processes such as fibrotic tissue stiffening (Asmani et al., 2018), to be recreated and observed directly on-chip; and measuring such phenomena *via* embedded sensors could offer crucial real-time insight into how biological systems evolve during homeostasis, pathology, and in response to candidate therapeutics (Soucy et al., 2019). Ideally, these real-time measurements would also be non-destructive to support repeated analysis of the same precious sample material; and would not interfere with the biological process itself (Wu et al., 2020; Danku et al., 2022). Direct measurement of transepithelial/transendothelial electrical resistance (TEER) across cell-generated barriers has emerged as an example of this, and is used to assess barrier integrity, drug toxicity, and transport phenomena (Park et al., 2019; Bossink et al., 2021) under physiologically-relevant insult and injury (van der Helm et al., 2019). This approach has been applied to probe on-chip models of gut (Henry et al., 2017), lung (Walter et al., 2016), skin (Alexander et al., 2018), retina (Chen et al., 2020), heart (Maoz et al., 2017), and the blood-brain barrier in response to shear stress (Tu et al., 2021).

Electrochemistry-based sensors have also emerged as key strategies in the context of real-time measurements, as they offer high measurement sensitivity, low limits of detection, and high selectivity, particularly when coupled with analyte-detection technologies (Adam Kratz et al., 2019). These approaches have been used to measure secreted analytes *via* antigen- (Lee et al., 2021), aptamer- (Shin et al., 2016; Tran et al., 2021) (Tran et al., 2021), enzyme- (Bavli et al., 2016), and bead- (Riahi et al., 2016) based binding assays. Ortega et al. (2019) recently used these approaches to measure real-time release of myokines from a muscle-on-a-chip, and demonstrated that tissue response to electrical stimulation specifically is highly transient and disappears within 30 min, further emphasizing the impact of incorporating real-time sensing on-chip.

### 2.2 Multiplexed measurements

Operating an organ-on-a-chip requires technical skill and ability, and failed experiments are common at this relatively early stage of the technology lifecycle. Hence, extracting multiple measurements from the same sample is crucially important in understanding the complex biological system emerging from disparate components (Picollet-D’hahan et al., 2021), but also providing sufficient return-on-investment for the system. Furthermore, the integrative nature of biology suggests that multimodal measurements are essential to capture disease processes and functional therapeutic activity (Ort et al., 2021). An excellent example of this is work by Zhang et al., who demonstrated an integrated platform of multiple organoid microbioreactors with integrated physical sensors to monitor pH, O2, and temperature; electrochemical sensors to measure protein biomarkers; and optical sensors to monitor organoid morphology, allowing them to combine qualitative and quantitative observations and capture holistic function-molecule relationships (Zhang et al., 2017a).

### 2.3 Spatial measurements

Despite the small volume of a typical OoC culture chamber, understanding the spatial distribution of features within that space is essential to comprehend the impact and evolution of biomarkers in physiological and pathological activity in diseases (Shin et al., 2021). For example, patterns of electrical activity in neural tissues are characteristic of disease state and can be measured *via* micropatterned multi-electrode arrays (MEA). These sensor arrays were used to be stimulate and measure spatial electrical activity in pre- and post-synaptic neuronal structures (Moutaux et al., 2018). A similar strategy applied to heart-on-a-chip platforms allowed Maoz et al. to integrate both MEA and TEER electrodes on a heart-on-a-chip platform, permitting real-time measurement of electrical activity across the cardiomyocyte cultures (Maoz et al., 2017).

## 3 Measuring mechanics-on-a-chip

Cell mechanics play a fundamental role in several biological processes, defining fate and function of cells and tissues. Several methods have been developed to quantify and characterize the mechanical microenvironment that cells live in, and have been designed to measure both cell-generated forces and mechanical properties of cells and tissues. Overviews of force measurement and mechanical characterization methods are extensively reviewed elsewhere (Polacheck and Chen, 2016; Roca-Cusachs et al., 2017; Nguyen and Kilian, 2020; Obenaus et al., 2020), and here we focus specifically on the opportunities and challenges that come with integrating these sensors into OoC and microtissue engineered models, and how real-time, multiplexed, and spatially-defined information about tissue function can be obtained.

**FIGURE 1:**
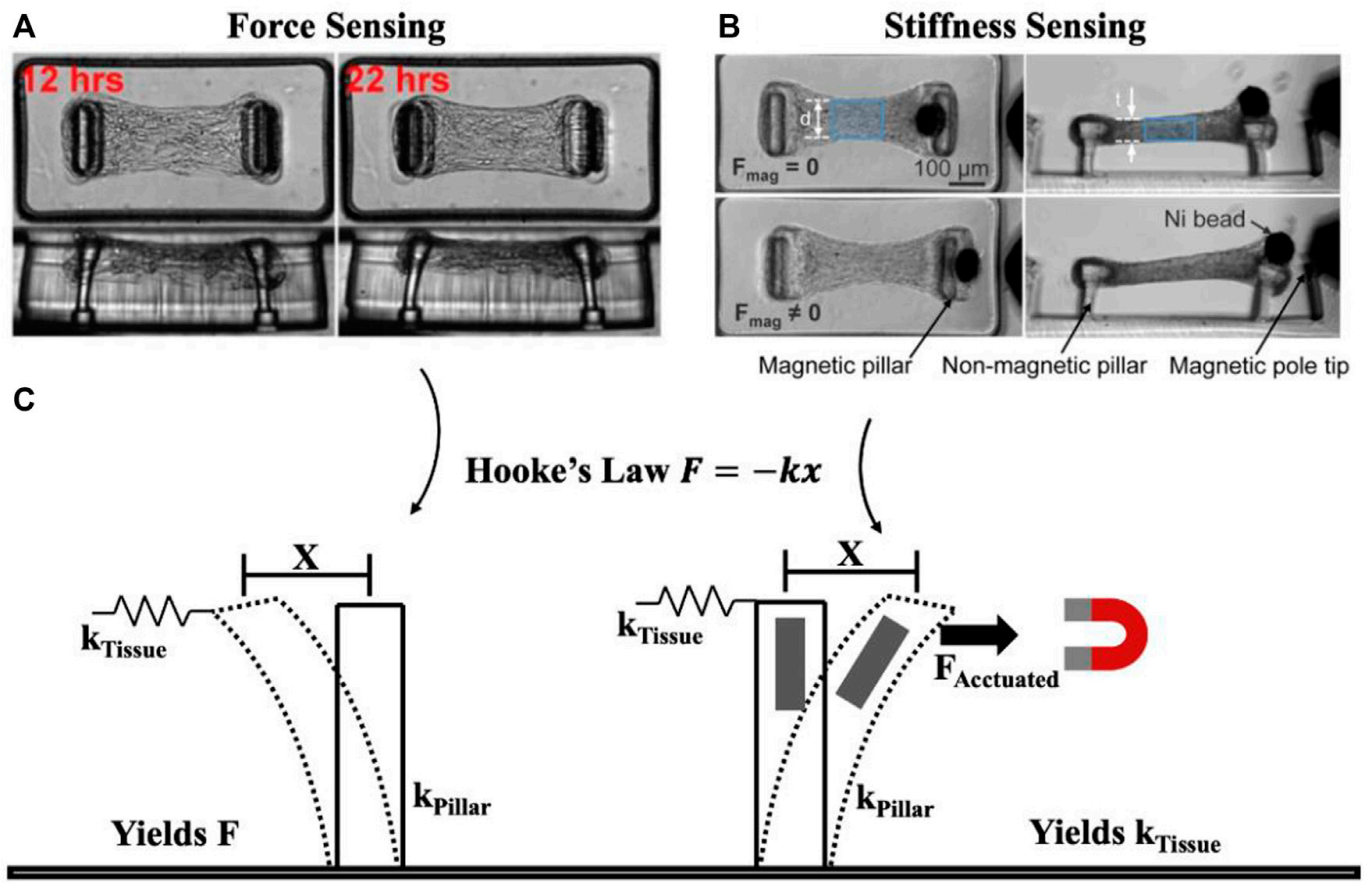
Simplified view of underlying principle behind force and mechanical property (e.g., stiffness) measurement. Tissue force sensing **(A)** and stiffness sensing **(B)** are both possible using micropillars. Considering pillars to behave as springs, force is measured based on pillar deformation *x* and pillar spring constant *k*_*Pillar*_ to yield force *F*, while stiffness is measured by pillar deformation *x* as a result of applied force *F*_*Actuated*_ to yield the spring constant *k*_*Tissue*_ **(C)**. Essentially, mechanical property measurement requires a form of actuation unlike force sensing. **(A)** Representative time course imaging of a contracting microtissue. Reprinted with permission from Legant et al. (2009), Proc. Natl. Acad. Sci. 106(25). 10097-10102 (2009). **(B)** Microtissue before (**F**_mag_ = 0) and after (**F**_mag_ ≠ 0) magnetic force is applied to stretch the tissue. Reprinted with permission from Zhao et al. (2013), Adv. Mat. 25(12): 1699-1705 (2013). Copyright 2013 John Wiley and Sons.

To meaningfully review the techniques used for measuring mechanics in OoC models, it is helpful to simplify the core principles underlying measurement techniques. To do this, we present a highly simplified view of mechanical characterization techniques, in which systems can be considered as a single spring with spring constant *k* which undergoes displacement *x* from an applied force *F* according to Hooke’s Law *F = −kx*. Measuring forces (*F*) requires knowing the stiffness of the deforming spring element, while measuring stiffness (*k*) requires applying a known force, and measuring the resulting displacement (Figure 1). While this linear, elastic simplification does not capture more complex mechanical behaviours such as viscoelastic or plastic deformations, this simple model can ultimately be extended towards these more challenging measurements by capturing the force, displacement, and stiffness (k) features as a function of time or stress to match the appropriate constitutive model. In the following sections, we highlight how this simple model has been used in OoC and microtissue analysis to improve our understanding of biological systems.

### 3.1 Force measurement

Cell-generated forces are known to play critical roles in biological development, stabilizing tissue structure, as well as determining tissue fate and function. Processes like cell migration, muscle contraction, wound healing, morphogenesis, and cancer progression have been found to be fundamentally governed by cell-generated forces, prompting the development of methods to quantify such forces. Measuring cell forces generally relies on relating cell-induced displacements of mechanically characterized substrates to the applied force. Therefore, techniques have been established to quantify forces spanning subcellular to tissue scales, in both 2D and 3D contexts, by measuring the force-controlled deformation of well-defined structures (Figure 1).

The most common technique to quantify contractile forces exerted by adherent cells pulling on a flat 2D surface of known stiffness, referred to as traction force microscopy (TFM) (Pelham and Wang, 1999; Munevar et al., 2001; Wang et al., 2001). Linearly elastic substrates are embedded with fiduciary markers, either as dispersed fluorescent microbeads (Oliver et al., 1998; Dembo and Wang, 1999; Maruthamuthu et al., 2011) or ordered micropatterned arrays (Balaban et al., 2001; Polio et al., 2012) to facilitate stress field computation. Tracking the displacement of these markers in response to cell-generated forces can then be used to obtain a displacement field. If the mechanical properties of the substrate are well known, this field can then be used to calculate the stresses generated by cells. Commonly used materials to make substrates with tunable stiffness include silicones (Lee et al., 1994), PDMS (Yoshie et al., 2018; Yoshie et al., 2019), and hydrogels (Pelham and Wang, 1999)**,** both of which are compatible with current organ-on-a-chip fabrication technologies (Campbell et al., 2021).

Continuous surfaces present a uniform adhesion profile to cells, and this may not replicate the adhesive profile present in many biological systems. To address this, micropillar arrays can be fabricated in silicone rubber with precise control over pillar dimensions and material stiffness. Tracking the deflection of these micropillars can then be used to calculate local stresses at that point (Tan et al., 2003), while independently manipulating the stiffness and adhesive profile presented (Fu et al., 2010). Cells can also be cultured as well-connected colonies on both flat (Trepat et al., 2009; Tambe et al., 2011; Serra-Picamal et al., 2012) and micropillar substrates (Rouredu et al., 2005; Ganz et al., 2006)**,** to understand both cell-cell and cell-substrate forces. Given that these silicone rubber-based fabrication methods are very similar to those commonly used in OoC design, these sensors can be readily integrated into microfluidic systems.

Cell-generated forces can also be measured in more physiologically relevant 3D contexts by embedding cells or tissues in engineered, natural, or composite matrices. As in two-dimensional systems, fiducial markers can be tracked in a 3D deformable matrix as they move due to forces generated by embedded single cells (Legant et al., 2010; Steinwachs et al., 2016; Malandrino et al., 2019) or microtissue constructs of varying geometries (Gjorevski et al., 2015; Leggett et al., 2020; Mark et al., 2020). As these methods are computationally intensive, an alternative strategy has emerged whereby well-defined structures are embedded within a tissue, and deformation of those structures can be used to calculate local stresses. Cantilevers surrounding an engineered microtissue (Figure 1A) (Legant et al., 2009; Thavandiran et al., 2013; Sakar et al., 2016; Chen et al., 2019) (Aoun et al., 2019) can be used to report tensional and compressive stresses at these peripheral sites. This broadly useful strategy can also be used to tune the mechanical features presented to tissues by manipulating tissue geometry (Asmani et al., 2018; Bose et al., 2019). In more recent and complex OoCs, this strategy has been used to measure stresses generated by activation of a neuromuscular junction on-chip (Vila et al., 2021), demonstrating the ability to make real-time, and multiplexed measurements in highly realistic engineered model systems.

Alternative structures to cantilevered pillars have also been used to measure forces on-chip. Silicone films of well-defined thickness will curl as cells cultured on their surface contract, and this approach has been used to study beating myocardial microtissues (Grosberg et al., 2011; Agarwal et al., 2013). Soft silicone pillars can be embedded within tissues to measure stresses during tissue contraction (Moraes et al., 2015). Wires with well-defined material properties can also be embedded at each end of contractile tissues, and the extent of their deflection is correlated with the applied force (Wang et al., 2019; Zhao et al., 2019). Co-culture wire systems have recently been used to study spatially patterned microtissues and effects on myocardial function (Zhao et al., 2019). Current wire-based OoC designs provide perfusable models by integrating a hollow fiber at the core of seeded tissues (Xie et al., 2020), while other designs guide tissue formation in self-assembling constructs (Portillo-Esquivel et al., 2020). In the perfusable hollow fiber model, the fiber walls are observed to contract and deform under the applied load of the tissue surrounding the fiber, highlighting the possibility of measuring tissue-generated forces on the fiber (Xie et al., 2020). For self-assembling tissue constructs, the wire provides an initial supporting structure, and deforms with the tissue during self-assembly, providing a readout of evolving contractility (Portillo-Esquivel et al., 2020). In both designs, mechanical forces can be suggested to play a role in defining overall tissue shape, which is known to regulate biological fate and function. Typical OoC platforms provide the necessary mechanical support to study tissues with varying geometries in 3D, highlighting a unique advantage of such systems. Thus, wire-based designs can further be utilized to study the effects of forces in varying tissue geometries and perfusable systems on-a-chip.

A key limitation in these techniques is that the measurement structure defines the tissue shape and dimensions, and thereby intrinsically changes the system being studied. To avoid this problem, several groups have recently developed dispersible cell-sized mechanosensors with sufficient compliance to resolve cellular scale forces and their spatial patterns within tissues. Oil microdroplets can be used to measure anisotropic forces (Lucio et al., 2017; Serwane et al., 2017), while hydrogel microdroplets can be used to measure isotropic forces like tension and compression (Dolega et al., 2017; Mohagheghian et al., 2018; Mongera et al., 2018; Lee et al., 2019). These sensors are easily embedded and mechanical properties are tuned for appropriate applications, from measuring applied compressive stresses on the order of kilopascals (Dolega et al., 2017) to cell-generated tensile forces (Lee et al., 2019). Fibrous matrices can also be used to resolve cell-scale measurements at the single cell state (Sheets et al., 2016) and in multicellular constructs (Ma et al., 2018; Wang et al., 2020) by deflecting under locally applied forces in the tissue. Since these types of sensors are incorporated within tissues, they can easily be integrated into OoC models to provide higher spatial resolution and multiplexed force readouts in real time.

### 3.2 Mechanical properties

Mechanical properties of tissues have been consistently proven to be a fundamental factor in cell behavior, tissue morphogenesis, and function (Guimarães et al., 2020), but defining the characteristics of an ideal measurement can be challenging. The length scale at which mechanical properties are measured in biological systems plays an important role as sub-cellular, cellular, and tissue-level measurements can produce distinctive results. Given the heterogenous nature of biological tissues, the precise location of the measurement can also have a considerable impact on the results. Finally, the mechanical model being used to describe a tissue may not adequately capture the tissues’ mechanical behaviour: tissues are often assumed to be linearly elastic and are evaluated by their elastic modulus (E), but more often exhibit time-dependent viscous properties which are much more challenging to measure (Huang et al., 2019). Despite these broad challenges, all methods require the application of a stress or strain on the material being tested, and this can be present some challenges in tightly regulated OoC systems.

Traditional materials characterization techniques of whole-tissue tensile and compression testing can be challenging to integrate with OoC systems, as the scale, enclosed nature, and constraining geometries in most OoCs are not compatible with this equipment. However, they can be applied to certain open systems in which the tissue can be more readily accessed. For example, micro tweezers allow compression testing to measure the bulk stiffness of a tissue (Jaiswal et al., 2017). Exposed surfaces can allow the use of conventional systems that resolve spatial tissue profiles. Conventional indentation tests can be applied at a range of length and depth scales, with indentation tips ranging from ∼10 µm to centimeters (Diez-Perez et al., 2010; Beekmans et al., 2017; Maccabi et al., 2018). Nanoindentation techniques such as atomic force microscopy (AFM) further miniaturize this concept and can measure mechanical properties in regions as small as a single cell (Wu et al., 1998; Oyen, 2013; Chen, 2014). Embedded sensors such as magnetic microparticles can measure the shear modulus of cells within a tissue using magnetic twisting cytometry (Kasza et al., 2011; Coughlin et al., 2013; Zhang et al., 2017b). Emerging technologies may also play a role in these studies, including sound-based acoustic elastography, which can be a minimal-contact strategy to measure mechanical properties by monitoring the surface wave speed, and this technique can be coupled with embedded stress sensors to produce quantitative results (Zhang and Greenleaf, 2007; Li et al., 2012; Kennedy et al., 2015). Brillouin microscopy, a form of optical elastography, uses the scattering of light from vibrational waves to determine the longitudinal modulus which can be related to other mechanical properties (Prevedel et al., 2019; Antonacci et al., 2020).

Each of these strategies require considerable peripheral equipment, and limit the dimensions and characteristics of the tissues being studied. Fortunately, several recent examples have embedded bulk mechanical characterization of microengineered tissues directly into the design of the OoC device. For example, MacQueen et al. (2012) integrated on-chip strain sensors to measure tissue compression in response to deformations applied by pneumatically-actuated compressing micro-platens. This system allowed mechanical characterization of microtissues embedded in a fluidically-controlled device. These systems allow for simultaneous and high-throughput measurement of tissue mechanical properties on-chip, but does require integration of complex electromechanical components for both micro-scale actuation and measurement. Alternatively, passive sensing structures can be used in combination with external actuation systems such as pneumatic- or magnetically-actuated components. For examples, cells adhering to micropillar force-sensing surfaces can be stretched at individual adhesions (Sniadecki et al., 2008) or as a whole (Mann et al., 2012) (Lam et al., 2012) by integrating magnetic nanowires, or stretching the whole array on a pneumatic device. Similarly, cantilevers used for measuring active 3D tissue contractility can be mounted with magnetic beads or bars (Zhao et al., 2013; Xu et al., 2015; Liu et al., 2016), on stretchable frames (Asmani et al., 2018) or on vacuum-actuated chambers to apply deformations to the tissues (Walker et al., 2020).

While actuated cantilevers consider the mechanical behaviour of whole tissues in real time, their spatial resolution is limited and cannot resolve variations at the single cell scale. To do so, sufficiently compliant, actuatable, and measurable sensors must be embedded within tissues. Magnetic ferrofluids were recently incorporated in deformable oil microdroplets, yielding actuatable microdroplets that have the dual purposes of measuring forces when not actuated and measuring stiffness when actuated by a magnetic field (Serwane et al., 2017). Incorporating such sensors inside tissues seeded in OoC platforms can further refine the spatial resolution of multiplexed measurements taken on a chip, providing both force and mechanical property readouts in real time. As a simpler alternative to avoid the experimental complexity of creating and manipulating a uniform magnetic actuating field, thermally actuated size-changing hydrogel beads have been dispersed within microtissues and used to measure stiffness at the length scale of individual cells (Proestaki et al., 2019; Mok et al., 2020). High-stiffness environments limit the expansion of these microgels when the temperature is dropped below 37°C, allowing users to measure the apparent stiffness at the site of sensor inclusion. While these systems have thus far only been used to measure spatially-defined mechanical properties in organisms, organs, and engineered microtissues, their optical addressability and minimal additional infrastructure requirements make them ideally suited to study OoC systems.

## 4 Perspective and conclusion

Although OoC platforms present a unique opportunity to understand transitions in realistic biological systems and have been popularized for over a decade, the study of mechanically-driven tissue evolution remains relatively limited, especially compared to advances in molecular, genomic, proteomic, and secretory analysis techniques. We believe that while molecular-based analysis techniques are comparatively straightforward to apply to OoCs, the need to both apply and measure forces and displacements do not lend themselves easily to the small volume, enclosed nature, and relatively low robustness of these technologies. Hence, developing new strategies to integrate mechano-sensors that directly address these issues will be of critical importance in establishing the time-resolved, multiplexed, and spatially-defined sensing capabilities that have been particularly impactful in conventional sensor-OoC integrations.

There are several use-cases in which mechanical sensors used in these contexts would be of critical importance. In diseases such as fibrosis and asthma, mechanical changes directly influence disease progression. Fibrosis is the progressive stiffening of tissue by the chronic accumulation of extracellular matrix (ECM), and has been associated with morbidity and mortality in disease affecting the lung, liver, kidney, and heart valve. Recreating the initial architecture and mechanical state of such tissues, the acute and chronic wounding and inflammation-driving stimuli, and the positive feedback cycle of tissue stiffening and cellular remodeling all require advanced OoC-tissue engineering strategies. Coupled with the broad impact of fibrosis-oriented diseases, and the very clear relationship between environmental mechanics and disease progression, tracking these changes *via* OoC sensors has already been shown to have great potential in drug screening (Asmani et al., 2018). However, the focal nature of this disease strongly suggests that spatially-resolved measurements coupled with physiologically realistic recreation of tissue insult can both help us understand the mechanical nature of disease progression, but also allow us to develop strategies to reverse these conditions, rather than halting disease progression. The slow progression of the disease will also require time-resolved measurements, in which small cumulative changes can be detected as they occur, which is particularly challenging to do in conventional cultures. Finally, the need to understand complex multimodal relationships such as tissue stiffness and oxygenation, strongly suggest that a time-resolved, multimodal, and spatially-defined knowledge of the tissue are essential to address this complex disease.

In contrast to the slow progression of fibrosis, diseases such as asthma involve enhanced force generation and stiffening of the airway smooth muscle cells, contributing to excessive narrowing of the airways and hyperresponsiveness in asthma. These changes occur within minutes, and understanding the capacity of the involved cells to contract, generate forces, and remodel the tissue may provide novel perspectives of the underlying mechanisms of asthma pathophysiology. Understanding these diseases will require high time-resolution measurements to both understand the effectiveness of potential therapies, and the factors that trigger attacks. These tools must therefore rapidly capture a large dynamic range of sensor readings, and will hence have distinctive characteristics over long-term sensors needed for fibrosis studies, highlighting the need to design and integrate application-specific sensors into OoC models.

**FIGURE 2:**
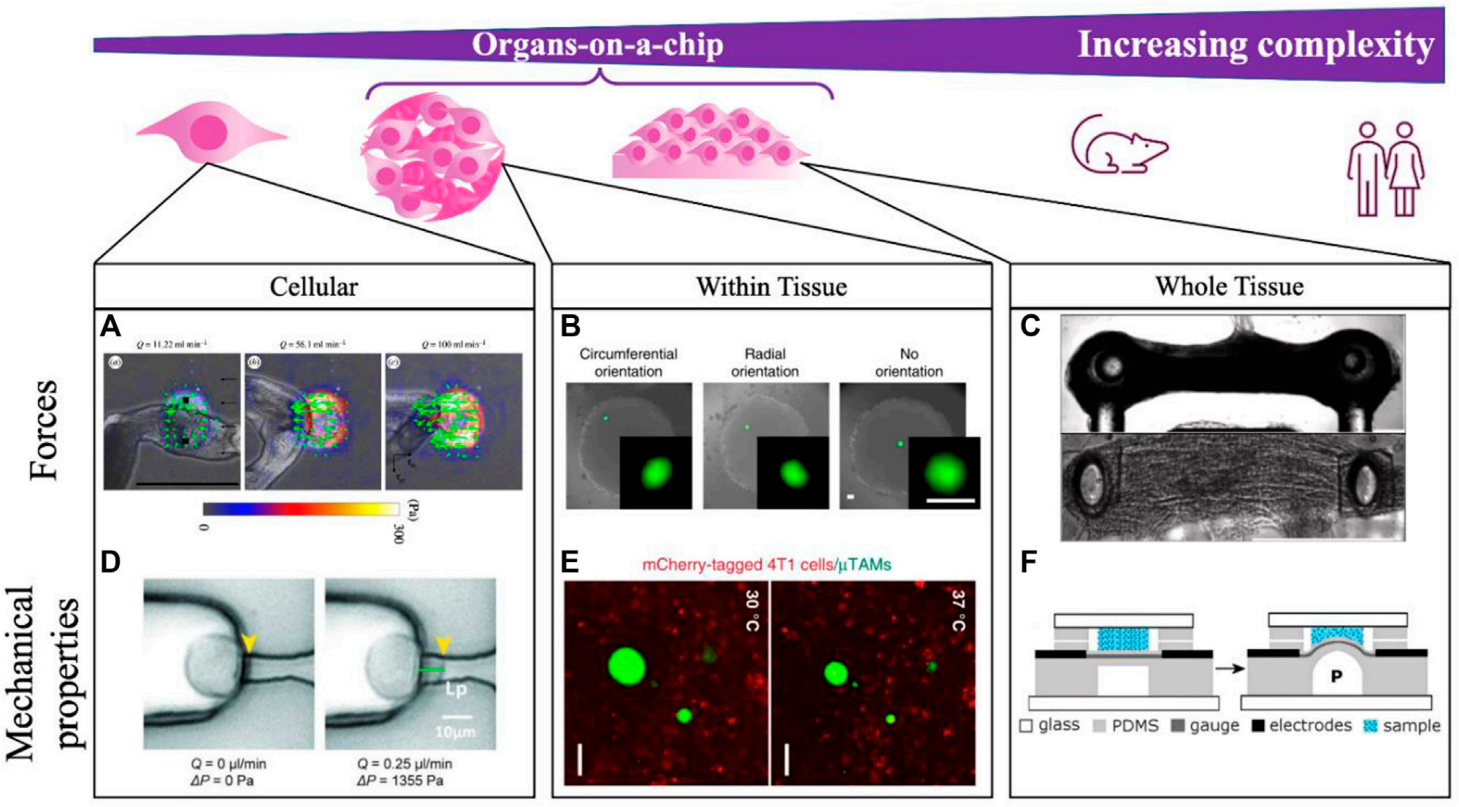
Integrating mechanical measurements on-a-chip can be done to study tissues spanning a range of complexities. Measurements are made at the single cell scale **(A,D)**, locally within self-assembled tissues **(B,E)**, and across whole dynamic tissues **(C,F)**. **(A)** *Schistosoma mansoni* generated forces are quantified by integrating TFM-on-a-chip and modulating a shear force applied to cells with flowrate *Q* in the microfluidic channel. Scale bar 500 μm. Reprinted with permission from Zhang et al. (2019), J. R. Soc. Interface 16: 20180675 (2018). Copyright 2018 The Royal Society (UK) **(B)** Within tissures, compliant hydrogel microdroplets deform under applied loads in a fibroblast multicellular spheroid, with bead orientation indicating the direction of applied cell-generated forces. Scale bare 50 μm. Reprinted with permission from Lee et al. (2019), Nat. Commun. **10**, 144 (2019). Copyright 2019 Author(s), licensed under a Creative Commons Attribution (CC BY) license. **(C)** Contractility of microtissues is measured according to the deflection of embedded pillars in neuromuscular-junction on-a-chip. Scale bar 500 μm. Reprinted from Vila et al. (2021), Biomat., 276:121033 (2021). Copyright 2021 Elsevier. **(D)** Mechanical properties can further be measured on-a-chip by locally aspirating and deforming the cell membrane with a known vacuum pressure applied through a microfluidic channel. Reprinted with permission from Lee and Liu (2014), Lab on a Chip 15 (1), 264–273 (2015). Copyright 2015 Royal Society of Chemistry (UK) **(E)** Within tissues, local stiffness measurements are made by incorporating thermoresponsive hydrogel beads, where the extent of bead expansion is correlated with surrounding tissue stiffness. Scale bar 50 μm. Reprinted with permission from Mok et al. (2020), Nat. Commun. **11**, 4757 (2020). Copyright 2020 Author(s), licensed under a Creative Commons Attribution (CC BY) license. **(F)** Whole tissue stiffness is quantified by applying a known pressure to the tissue through a bulging device integrating on-a-chip. Reprinted with permission from MacQueen et al. (2012), Lab on a Chip 20 (12), 4178 (2012). Copyright 2012 Royal Society of Chemistry (UK).

In addition to understanding the application, it is also helpful in both the mechanical sensor design and development of the scientific application to precisely conceptualize the scale at which the measurement is being made (Figure 2). Measurements at the sub-cellular, cellular, and tissue length scales are all possible with various OoC formats, but address very different biological questions. For example, our group has consistently found that despite tissues having well-defined global patterns of force generation (Lee et al., 2019) and mechanical stiffness (Mok et al., 2020), designing cellular-length scale sensors reveals considerable heterogeneity in both force and stiffness, likely due to the highly-local action of specialized cells within a tissue construct, and the distinct structures being interrogated at various length scales. Different technological approaches will likely be required for each of these length scales (Figure 2), with different design approaches. For example, sub-cellular measurements of force using traction force microscopy-based approaches may best be accomplished by simply incorporating fiducial particles into a soft substrate, here used to quantify cellular contractility while exposing the cells to shear flow in a microfluidic channel (Jang et al., 2019; Zhang et al., 2019). These stresses are highly diverse, and cannot be easily integrated to capture stresses that drive disease-specific phenotypes. Measuring cellular level forces requires those fiducial particles to have defined mechanical properties and capabilities, but cannot capture highly focal applications of force that may be relevant for certain biological processes. Measuring tissue-level forces requires anchored pillars that limit tissue shape, but provide sufficient resistance to the movement that forces can be measured (Vila et al., 2021).

Several core challenges exist in designing and applying these sensors to OoCs that remain to be addressed. First, whether a measurement system interferes or modifies the biological system being studied will remain an open question and must be carefully answered on a case-by-case comparative basis. For example, whether soft hydrogel particles included in a tissue to measure local force (Lee et al., 2019) change the function of that tissue remains an open question. While steps can be taken to coat the particles with cell- and tissue-like materials, the simple inclusion of a mechanically distinct region within the tissues may itself alter the biological system, and must be validated carefully against control conditions for the variable of interest. Second, the measurement system must be compatible with both the material properties and processing attributes of the OoC system. For example, despite being similar materials, integrating silicone micropillars into standard silicone devices presents significant challenges in releasing the pillars without collapsing them due to surface tension effects, and bonding surfaces *via* plasma oxidation without changing the surface mechanical properties of the sensor. Third, OoC platforms are often touted as scalable alternatives to animal models, and integrating mechanical sensors significantly increases their complexity, and hence susceptibility to failure. This is particularly important in applications that require high-throughput analyses and experimentation such as drug screening, and developing these sensing systems to be sufficiently robust for such applications constitutes an important design challenge for the future.
